# Effect of Different Accumulative Temperate Zones in Heilongjiang on Glycine Soja Metabolites as Analyzed by Non-Target Metabolomics

**DOI:** 10.3390/molecules28083296

**Published:** 2023-04-07

**Authors:** Guofeng Bao, Liqiang Mu, Ying Wang

**Affiliations:** 1 College of Forestry, Northeast Forestry University, Harbin 150040, China; bgf19850124@nefu.edu.cn; 2 National Coarse Cereals Engineering Research Center, Heilongjiang Bayi Agricultural University, Daqing 163319, China; wangying@byau.edu.cn

**Keywords:** glycine soja, accumulated temperature zone, amino acid, Heilongjiang, differential metabolite

## Abstract

To study the effect of growth temperature on the nutritional components and metabolites of the wild soybean (*Glycine soja*), we analyzed the nutritional components and metabolic gases of the wild soybean in six accumulated temperature regions of the Heilongjiang Province, China, by gas chromatography–time-of-flight mass spectrometry (GC-TOF-MS). A total of 430 metabolites, including organic acids, organic oxides, and lipids, were identified and analyzed using multivariate statistical analysis, orthogonal partial least squares discriminant analysis, principal component analysis, and cluster analysis. Eighty-seven metabolites significantly differed in the sixth accumulated temperature region compared with the other five accumulated temperature regions. The 40 metabolites (such as threonine (Thr) and lysine (Lys)) were found to be elevated in soybeans from the sixth accumulated temperature zone compared with the other five accumulated temperature zones. Through analyzing the metabolic pathways of these metabolites, amino acid metabolism had the greatest influence on wild soybean quality. The results of the amino acid analysis were consistent with those of the GC-TOF-MS and showed that amino acids in wild soybeans from the sixth accumulated temperature zone significantly differed from those of the other zones. Threonine and lysine were the main substances driving these differences. The growth temperature affected the type and concentrations of metabolites in wild soybeans, and the GC-TOF-MS analysis of the effect of growth temperature on wild soybean metabolites was shown to be feasible.

## 1. Introduction

Wild soybean (*Glycine soja Sieb. et Zucc.*), also known as the horse seed bean or black bean, is the ancestor of the cultivated soybean Glycine max [1]. Wild soybeans have desirable properties, such as being light-loving, humidity-tolerant, shade-tolerant, drought-resistant, disease-resistant, and barren-resistant [2]. They grow especially well on saline-alkali soils with a soil pH of 9.18–9.23 and can survive winter at −41 °C [3]. With their strong salt-alkaline and cold resistance characteristics, wild soybeans have unique genetic advantages and are important germplasm resources for improving the traits of cultivated soybeans and simultaneously have ecological significance for maintaining species diversity [4]. Wild sojas are distributed across all of China except the Xinjiang, Qinghai, and Hainan Provinces [5]. As the Heilongjiang Province is located in the high latitude region of China, its climate conditions are complex and temperatures vary greatly [6]. However, the soil is fertile and pollution-free and the concentrations of trace elements are the best in the country. As a result, the quality of wild soybean resources that grow naturally in this black soil is higher.

Cultivated soybean is an important dual-use crop, which is an important source of plant protein and plant oil, and the northeast region is the main production area of cultivated soybean [7]. With the continuous improvement of people’s living standards, more attention is focused on increasing the quality of soybeans and their by-products. Product quality is reflected in the form of crop quality, which is the result of the combined action of the genetic characteristics of a variety and environmental conditions. For a specific genetic basis, the role of the environment is very important. Previous studies on the relationship between environmental factors and crop quality have focused on light, temperature, moisture, carbon dioxide, cultivation system, and other factors [8,9]. In 2017–2021, breeders have increased their efforts to develop the sixth temperate zone, which is also emerging as an important soybean-growing region; this shows that Heilongjiang has started to increase the breeding of varieties in the low accumulated temperature zone [10]. Many researchers have examined the quality of wild soybeans, majorly focusing on the characteristics of wild soybean quality indicators, the physiological mechanisms of quality formation, and the genetic characteristics of quality traits [11,12]. However, there are few reports on the differences in the metabolites of wild soybeans from different accumulative temperature zones. The Heilongjiang Province spans 14 longitudes from east to west and 10 latitudes from north to south. Its geographical position is the northernmost point in China. It has a cold climate and the shortest frost-free period. However, what are the effect of accumulated temperature in different accumulated temperature zones on the quality of wild soybean in Heilongjiang Province? The different metabolites among these soybeans need to be further studied and explored. Therefore, based on previous studies, in this experiment, wild soybeans from different accumulated temperature zones in the Heilongjiang Province were selected for metabolomics research, as the determining metabolic differences of wild soybeans originating from different areas can contribute to improving the cultivation of high-quality protein soybean varieties and cultivated soybean germplasm resources.

## 2. Results

### 2.1. The Main Nutritional Components

The nutritional contents of wild soybeans from different accumulated temperature zones are shown in Table 1. The crude protein (CP) contents increased gradually with the increase in the accumulated temperature zone except in the fourth accumulated temperature zone, which may be due to the influence of different accumulated temperatures on the CP content in the crop growth period. The amounts of crude fat (EE) content and CP content of the wild soybeans were contrasting, with the EE content decreasing as the accumulated temperature zone; this result was the same as that of cultivated soybeans. The crude fiber, crude ash (CA), and nitrogen-free extract (NFE) of the wild soybean protein were not affected by the accumulated temperature zone.

### 2.2. The Amino Acid Composition

Table 2 summarizes the types and concentrations of amino acids in wild soybeans from different accumulated temperature zones. As shown in Table 2, the amino acid contents of the wild soybeans from each accumulated temperature zone were different. The amino acid contents of wild soybeans from the sixth accumulated temperature zone was notably higher than those from the other accumulated temperature zones. This result may be because the average temperature in the sixth accumulated temperature zone is lower and has a dark brown soil, and as wild soybeans have strong cold resistance and can thrive in black and newly accumulated soils, they are able to develop excellent characteristics in this zone. This result is consistent with the highest protein contents occurring in wild soybeans from the sixth accumulated temperature (Table 1).

The amino acid analysis of the wild soy protein isolates also showed that the cysteine (Cys) and methionine (Met) contents were low, which is mainly due to the loss of these two amino acids caused by acid hydrolysis in the amino acid determination. All nine essential amino acids were much higher than the recommended values from the FAO, which indicates that wild soybean protein isolates are plant proteins with rich nutritional and development value.

### 2.3. Gas Chromatography-Time of Flight Mass Spectrometry

**Figure 1:**
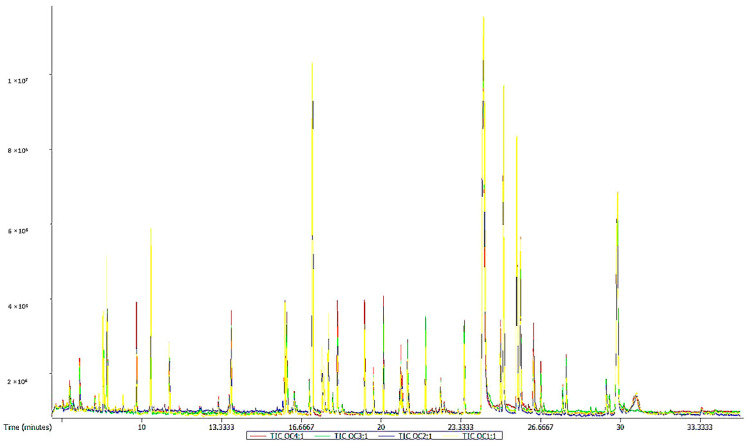
TIC overlay of all quality control (QC) samples.

Using the ChromaTOF software and using the LECO-Fiehn Rtx5 database, 430 metabolites were analyzed. The TIC stack diagram of all quality control (QC) samples are shown in Figure 1, which suggests that the peak area and retention time of the TIC of the QC samples overlap well, indicating that the results of the instrument were stable and the analysis by the instrument was reliable. The results showed that 255 metabolites were accurately identified in the sixth accumulated temperature zone, whereas 397 metabolites were annotated in the other five accumulated temperature zones. These metabolites were mainly amino acids, organic acids, organic oxides, lipids, and lipid-like molecules.

### 2.4. OPLS-DA Result

**Figure 2:**
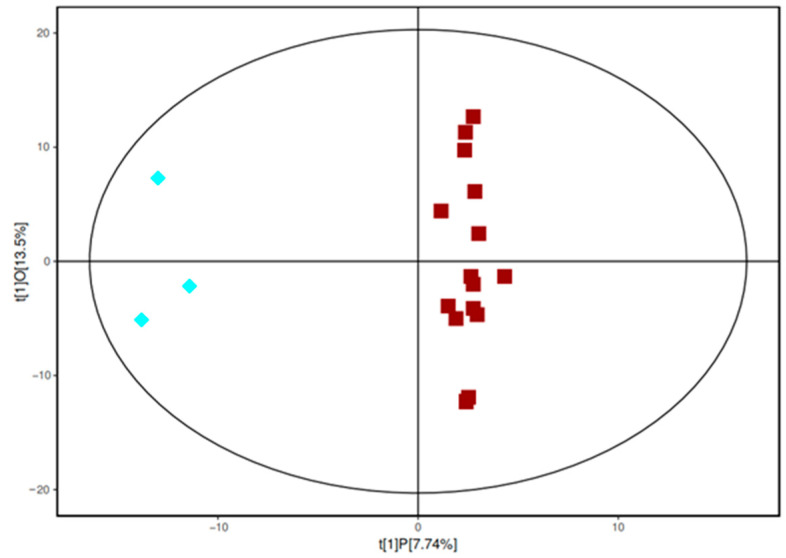
The OPLS-DA model of the sixth accumulative temperature zone (blue diamonds) and the other accumulative temperature zone samples (red squares).

The results of the principal component analysis showed that no significant difference was present in metabolism between samples from the sixth accumulated temperature zone and those from the other accumulated temperature zones. Through OPLS-DA analysis, orthogonal variables that were not related to categorical variables in the metabolites were filtered out, and non-orthogonal and orthogonal variables could be analyzed separately, thus resulting in the highly reliable intergroup differences of the metabolites and the correlation degree information of the experimental groups. Figure 2 shows the OPLS-DA score diagram of the sixth accumulated temperature zone and the other five accumulated temperature zones. All samples were within the confidence intervals. The difference between the two groups was evident (Figure 2). No overlap was present between the samples and the separation was high. The clustering of samples from the first to fifth accumulative temperature zones was strong, which may be because wild soybeans from these different accumulative temperature zones were affected by different growth temperatures, but most of their metabolites were the same and the metabolite composition of these samples was similar, resulting in the sample points being close or clustered.

**Figure 3:**
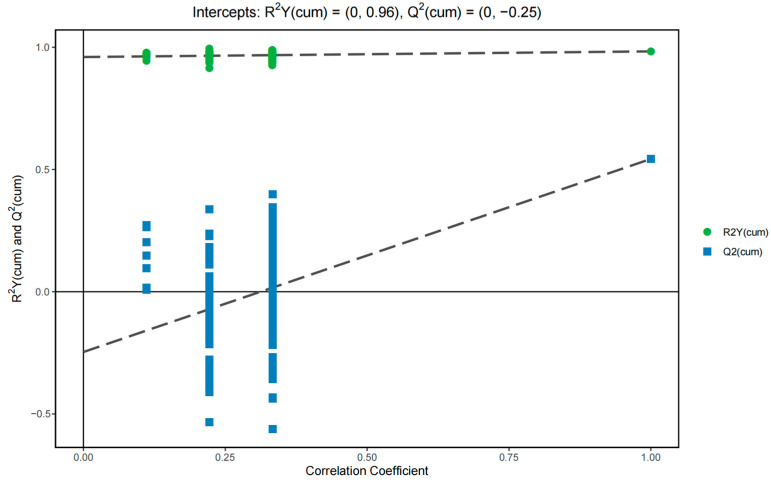
Permutation test results of the OPLS-DA model of the sixth accumulative temperate zone against other accumulative temperate zones.

Figure 3 shows the permutation test results of the OPLS-DA model. Each group of models had two principal components. The cumulative values were R^2^X = 0.213, R^2^Y = 0.983, and Q^2^ = 0.543. Model R^2^Y was very close to 1, which demonstrated that the established model conformed to the real situation of the sample data. The original model can better explain the difference between the two groups of samples. Simultaneously, with the decrease in the permutation retention, the proportion of the permutation Y variable increased, whereas the Q_2_ of the stochastic model decreased gradually. This result showed that the original model had good robustness and no over-fitting phenomena were present.

### 2.5. Mining and Identification of Differential Metabolites

Differential metabolites were identified if the variable projection importance of principal component 1 of the OPLS-DA model was greater than 1 and the *p* value of the *t* test was ≤0.05. As shown in Table 3, 87 differential metabolites were screened out after comparing the sixth accumulated temperature zone with the other five accumulated temperature zones, which mainly included amino acids, sugars, fatty acids, organic acids, polyols, and other secondary metabolites. Amino acids were the main factors affecting differences in the nutritional composition of wild soybeans from different zones. Among the 87 differential metabolites, 40 metabolites (such as threonine (Thr) and lysine (Lys)) were found to be elevated in soybeans from the sixth accumulated temperature zone compared with the other five accumulated temperature zones. The results showed that wild soybeans from the sixth accumulative temperature zone differed greatly in their amino acid composition and content compared with wild soybeans from the other five accumulative temperature zones. The concentrations of the other 47 metabolites in wild soybeans from the sixth accumulated temperature zone were lower than those in the other five accumulated temperature zones. Additionally, the contents of the differential metabolites varied greatly between different samples, which shows that the metabolism of the wild soybean was highly variable. The metabolites were significantly affected by growth temperature, indicating that the wild soybean metabolites carried information specific to their accumulated temperature zone and that the differential metabolites can be used as the basis for distinguishing between accumulated temperature zones.

### 2.6. Cluster Analysis of the Differential Metabolites

**Figure 4:**
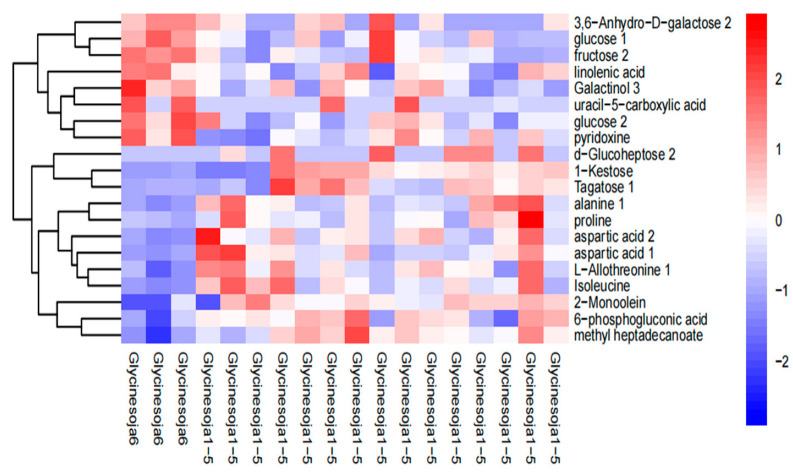
Hierarchical cluster heatmap of the metabolites.

Figure 4 shows the hierarchical cluster thermogram of the total metabolites in the six accumulated temperature zones. The highly expressed substances were amino acids, such as alanine, aspartic acid, and glycine, which is consistent with the results in Table 3. The left side of the figure represents the sixth accumulated temperature zone and the right side represents the other five accumulated temperature zones. The sixth accumulated temperature zone was a single sample with a significant clustering effect, whereas the other five accumulated temperature zones, as a whole, displayed no significant clustering effect, which demonstrates that the metabolites of wild soybeans from different accumulated temperature zones contain information about their accumulated temperature zone. Furthermore, distinguishing the accumulated temperature zones of wild soybeans is feasible using their metabolite differences.

### 2.7. Metabolite Pathway

**Figure 5:**
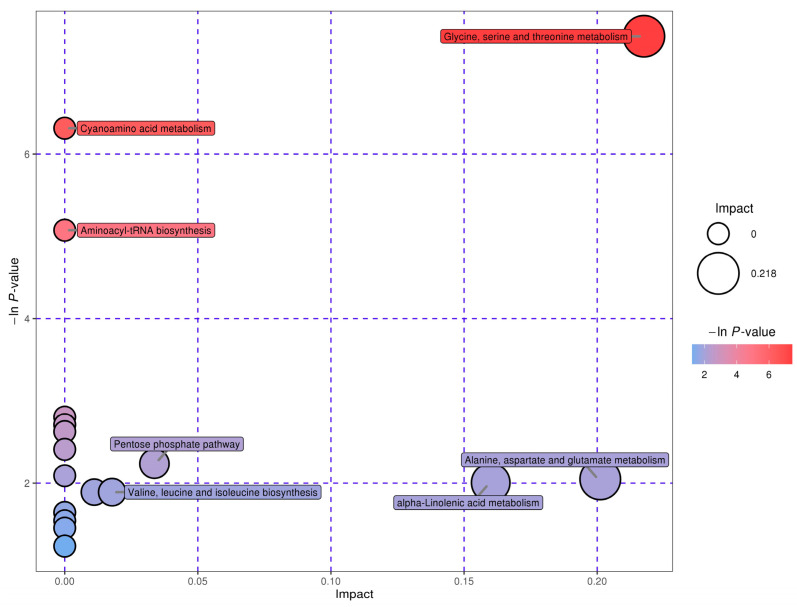
Factors affecting metabolic pathways.

The MetPA database was used to analyze the metabolic pathways of the differential metabolites between groups under the conditions of *p* value = 1 after false positive correction. Through metabolic pathway concentration and topological analysis, the metabolic pathways that may be biodisturbed were identified. Figure 5 shows the metabolic pathway influencing factor map. Each point represents a total of 20 points in a metabolic pathway map. These 20 metabolic pathways can be found in the KEGG database, which includes 10 amino acid metabolic pathways. Both synthetic pathways and decomposition pathways are numbered 1–10 in Table 4. A total of 49 differential metabolites involved in these metabolic pathways are shown in Table 5.

Table 5 shows that these substances are the hubs connecting each pathway at the nodes of the metabolic pathway network. Amino acid metabolic pathways comprise half of all the retrieved metabolic pathways.

## 3. Discussion

The average temperature is the main climatic factor affecting the phenological period of most soybeans [13]. The differences between the accumulated zones led to different qualities of wild soybean. This study confirmed that there are significant differences in metabolites of the same variety of wild soybean growing in different accumulated zones. The analysis results showed that among the 87 different metabolites identified in this study, 40 metabolites, such as Thr and Lys, were higher in soybeans from the sixth accumulated temperature zone than in the other five accumulated temperature zones. Threonine and Lys are essential amino acids for the human body. Therefore, the amino acid composition and the contents of wild soybeans in the sixth accumulated temperature zone are different from those in the other five accumulated temperature zones.

Amino acids are the basic components of proteins, and these substances are at the nodes of metabolic pathways and are the hubs connecting these pathways. Half of all the metabolic pathways identified in this study involved amino acids. The nutritional value of wild soybeans is mainly reflected in their amino acid contents, and wild soybeans are rich in nine essential amino acids required by the human body: leucine (Leu), phenylalanine (Phe), valine (Val), Thr, isoleucine, tyrosine, Lys, cysteine (Cys), and methionine (Met), which are much higher than the recommended values from the FAO. Among these, Leu had the highest concentration. Leucine can enhance immunity, activate human cells, and resist harmful bacteria and microorganisms. Isoleucine, Leu, Thr, Phe, Lys, Val, and Met are essential amino acids that are also important contributors to the nutritional value of wild soybeans.

It was found that amino acid was the main metabolic pathway of wild soybean in different accumulated temperate zones in Heilongjiang Province. Lin H et al. also identified 169 differential metabolites from soybeans, which were mainly involved in key pathways, such as amino acid biosynthesis and catabolism, lipid oxidation, and secondary metabolite accumulation [14]; amino acid was the main pathway; and environmental factors had a great impact on the metabolism of wild soybean. Some studies have found that amino acid metabolites, such as proline, is also able to act as osmotic factors in plants to improve survival when exposed to stressors including drought and salt stress [15,16]. The differences of temperature, environment, and other factors in different accumulated temperate zones in Heilongjiang Province lead to significant differences in their metabolites. In addition, amino acid metabolism and lipid metabolism were reported to play key roles in the drought resistance of different soybean varieties [17]. In summary, the metabolites of different accumulated temperate zones in Heilongjiang Province mainly concentrated on amino acids. Amino acids are not only important nutritionally but are also regulatory factors involved in adapting to local environmental conditions.

## 4. Materials and Methods

### 4.1. Materials and Chemicals

For this study, wild soybeans were collected in 2019 from the first accumulated temperature zone (Daqing city), the second accumulated temperature zone (Jiamusi city), the third accumulated temperature zone (Muling city), the fourth accumulated temperature zone (Yichun city), the fifth accumulated temperature zone (Heihe city), and the sixth accumulated temperature zone (Greater Higgnan Mountains.). These six accumulated temperature zones are beneficial to northeast China and were identified by Professor Mu Liqiang of the Northeast Forestry University. The plants with good growth and similar height were selected, and some mature seeds of good appearance and with no infections, diseases, or pests were cut and stored in air-permeable bags. Three samples were collected from each accumulated temperature zone, totaling 18 samples.

Chromatographic grade methanol was purchased from the CNW Technology Company of Germany (Germany). 3-Benzoylpyridine(Adamas) was purchased from Sigma-Aldrich Co. (Shanghai, China). Methoxyamine salt was purchased from Zhejiang Huafang Pharmaceutical Co., Ltd. (Tai Zhou, China). Ribonitol was purchased from Sigma-Aldrich Co. (Shanghai, China). BSTFA was purchased from Regis Technologies, Inc. (Wilmington, DE, USA). Saturated fatty acid methyl esters were purchased from Dr. Ehrenstorfer (Augsburg, Germany). Pure water was purchased from Watsons of China (Harbin, China).

### 4.2. A Breakdown of the Division of the Heilongjiang Temperate Zone

A breakdown of the division of the Heilongjiang temperate zone are shown in Table 6 [18].

### 4.3. Determination of Basic Nutrients

Rules for agricultural seed testing: the determination of moisture content (GB/T 3543.6-1995); the determination of protein in foods (GB/T 5009.5-2016); the determination of fat in foods (GB/T 5009.6-2016); the determination of crude fiber in plant foods (GB/T 5009.10-2003); and the determination of ash in food (GB 5009.4-2016).

Determination of dry matter [19]:

Dry matter (%) = 100-Moisture%

Method for calculation of nitrogen-free extract [19]:

Nitrogen free extract (%) = Dry matter% − (Crude protein% + Crude fat% + Crude fiber% + Crude ash%)

### 4.4. Analysis of Amino Acid Composition of Wild Soybean Protein Isolates

The amino acid determination method was as follows: a certain mass of the protein sample was added to 6 mol/L of hydrochloric acid, hydrolyzed at 110 °C for 24 h, then filtered and evaporated. The residue was dissolved in citric acid buffer solution with a pH 2.2, and Hitachi HITACHI835 -50 automatic amino acid analyzer was used for analysis.

### 4.5. Metabolite Extraction

The collected wild soybean samples with pods were air dried until the seeds cracked, after which the seeds were threshed naturally. After preliminary hulling, wild soybeans with full grains and no pests were selected and stored at −80 °C.

Derivation processing: 20 ± 1 mg of the sample was placed in a 2 mL EP tube, then 500 μL of pre-cooled extraction solution (methanol: chloroform volume ratio = 3:1) was added. Next, 10 μL of ribitol was added and was vortexed for 30 s. Porcelain beads were added and the samples was processed in a 45 Hz grinder for 4 min and the ultrasonic for 5 min (in an ice water bath). The samples were centrifuged at 4 °C and 12,000 rpm for 15 min. Subsequently, 100 μL of the supernatant was carefully pipetted into a 1.5 mL EP tube, while 40 μL of each sample was mixed to form a QC sample. The metabolites was dried in a vacuum concentrator. After evaporation in a vacuum concentrator, 50 μL of methoxyamination hydrochloride (20 mg/mL in pyridine) was added and then incubated at 80 °C for 30 min, then derivatized by 70 μL of BSTFA regent (1% TMCS, *v*/*v*) at 70 °C for 1.5 h. Gradually cooling samples to room temperature, 5 μL of FAMEs (in chloroform) was added to the QC sample. All samples were then analyzed by gas chromatograph coupled with a time-of-flight mass spectrometer (GC-TOF-MS) [20].

### 4.6. Computer Detection

Agilent 7890 gas chromatography-time-of-flight mass spectrometry (GC-TOF-MS) was equipped with an Agilent DB-5MS capillary column (30 m × 250 μm × 0.25 μm, J & W Scientific, Folsom, CA, USA). The GC-TOF-MS specific analysis conditions are provided in Table 7.

### 4.7. Statistical Analysis

Raw data analysis, including peak extraction, baseline adjustment, deconvolution, alignment, and integration [21], was finished with Chroma TOF (V 4.3x, LECO) software and LECO-Fiehn Rtx5 database was used for metabolite identification by matching the mass spectrum and retention index. Finally, the peaks detected in less than half of QC samples or RSD > 30% in QC samples was removed [22].

## 5. Conclusions

Based on GC-TOF-MS and other techniques, the nutritional components and metabolomics of wild soybeans from different accumulated temperature areas in the Heilongjiang Province were analyzed. The results showed that the different metabolites of wild soybean were present in the samples from different accumulative temperature zone in Heilongjiang. Wild soybean metabolite results revealed that 430 metabolites were identified and 87 differential metabolites were observed in the sixth accumulative temperature zone, while 40 metabolites (such as threonine (Thr) and lysine (Lys)) were found to be elevated in soybeans from the sixth accumulated temperature zone compared with the other five accumulated temperature zones. The results of different metabolite hierarchical clustering showed that the metabolites of different accumulative temperature zones were mainly expressed in amino acids, including alanine, aspartic acid, and glycine. Additionally, the signaling pathways of the differential metabolites also confirmed that their metabolic differentials were dominated by amino acid metabolisms, as they included 10 amino acid metabolic pathways (amino acid synthesis and catabolic pathways).

## Figures and Tables

**Table 1:** Analysis of nutritional components (mean ± standard deviation) of wild soybeans from different accumulated temperature zones.

Accumulated Temperature Zone	Nutritional Components					
	Dry Matter (DM)	Crude Protein (CP)	Crude Fat (EE)	Crude Fiber (CF)	Crude Ash (CA)	Nitrogen Free Extract (NFE)
First accumulated temperature zone	87.55 ± 1.02 a	41.95 ± 1.11 b	19.03 ± 0.42 a	4.1 ± 0.05 a	4.1 ± 0.08 a	18.37 ± 0.44 a
Second accumulated temperature zone	87.59 ± 1.13 a	43.27 ± 1.23 b	19.11 ± 0.21 a	3.8 ± 0.07 b	3.9 ± 0.07 b	17.51 ± 0.67 b
Third accumulated temperature zone	87.62 ± 0.87 a	45.65 ± 0.79 b	16.56 ± 0.47 b	4.2 ± 0.06 a	3.8 ± 0.06 b	17.41 ± 0.32 b
Fourth accumulated temperature zone	87.49 ± 0.56 a	45.04 ± 0.94 b	17.95 ± 0.29 b	3.7 ± 0.08 b	3.9 ± 0.07 b	16.90 ± 0.17 b
Fifth accumulated temperature zone	87.29 ± 0.93 a	46.15 ± 0.69 a	16.56 ± 0.33 b	3.8 ± 0.07 b	4.1 ± 0.08 a	16.68 ± 0.28 b
Sixth accumulated temperature zone	87.66 ± 0.79 a	46.65 ± 0.78 a	16.17 ± 0.28 b	3.6 ± 0.08 b	4.1 ± 0.05 a	17.14 ± 0.39 b

Different lowercase letters indicate a significant difference between treatments at the *p* < 0.05 level.

**Table 2:** Amino acid contents of wild soybean protein isolates.

Accumulated Temperature Zone	Amino Acid Content (g/kg)								
	Leucine (Leu)	Phenylalanine (Phe)	Valine (Val)	Threonine (Thr)	Isoleucine (Ile)	Tyrosine (Tyr)	Lysine (Lys)	Cysteine (Cys)	Methionine (Met)
First accumulated temperature zone	0.285 ± 0.07 b	0.275 ± 0.13 a	0.232 ± 0.06 b	0.187 ± 0.03 b	0.165 ± 0.08 b	0.138 ± 0.09 a	0.121 ± 0.08 b	0.072 ± 0.12 a	0.018 ± 0.02 b
Second accumulated temperature zone	0.333 ± 0.13 a	0.264 ± 0.04 b	0.245 ± 0.07 b	0.176 ± 0.07 b	0.173 ± 0.03 a	0.109 ± 0.08 b	0.114 ± 0.07 b	0.052 ± 0.07 b	0.017 ± 0.02 b
Third accumulated temperature zone	0.295 ± 0.14 b	0.231 ± 0.03 b	0.254 ± 0.04 a	0.168 ± 0.07 b	0.166 ± 0.12 b	0.117 ± 0.03 b	0.098 ± 0.06 b	0.047 ± 0.04 b	0.026 ± 0.05 a
Fourth accumulated temperature zone	0.272 ± 0.09 b	0.284 ± 0.15 a	0.242 ± 0.08 b	0.169 ± 0.09 b	0.179 ± 0.07 a	0.101 ± 0.05 b	0.091 ± 0.03 b	0.067 ± 0.07 a	0.015 ± 0.03 b
Fifth accumulated temperature zone	0.368 ± 0.14 a	0.258 ± 0.06 b	0.231 ± 0.05 b	0.206 ± 0.02 a	0.177 ± 0.06 a	0.120 ± 0.04 b	0.119 ± 0.11 b	0.059 ± 0.09 b	0.036 ± 0.03 a
Sixth accumulated temperature zone	0.389 ± 0.15 a	0.285 ± 0.17 a	0.253 ± 0.07 a	0.235 ± 0.03 a	0.165 ± 0.07 b	0.122 ± 0.02 b	0.168 ± 0.17 a	0.047 ± 0.08 b	0.018 ± 0.01 b

Different lowercase letters indicate a significant difference between treatments at the *p* < 0.05 level.

**Table 3:** Different metabolites of wild soybeans from the sixth accumulative temperature zone compared with wild soybeans from the other five accumulative temperature zones.

Serial Number	Metabolites	Matching Degree	Retention Time	Mass–Nucleus Ratio	Multiple	LOG_Fold Change
1	Threonine	615	24.9858, 0	191	4.034738444	2.012475152
2	Lysine	513	16.1544, 0	158	2.74526287	1.456944299
3	Galactinol	845	26.3387, 0	204	1.69245982	0.759121584
4	Fructose	941	17.6132, 0	103	1.551154488	0.633342379
5	Lactose	701	24.5229, 0	204	1.536435894	0.619587573
6	Threonic acid	879	14.056, 0	73	1.516431011	0.600679865
7	Elaidic acid	848	20.8699, 0	117	1.508722588	0.593327558
8	L-Malic acid	905	13.1961, 0	73	1.415874215	0.501693104
9	Tyrosine	529	17.745, 0	179	1.403227472	0.488748898
10	Epicatechin	852	25.8145, 0	179	1.337082019	0.419087966
11	Gluconic lactone	835	17.6669,0	129	1.32308485	0.403905586
12	Glucose	944	17.779, 0	160	1.316534485	0.396745312
13	Isocitric acid	523	16.9813, 0	71	1.31555597	0.395672629
14	Pyruvic acid	735	7.24774, 0	174	1.292478361	0.370140127
15	Linolenic acid	800	20.8693, 0	93	1.289148146	0.366418065
16	Erythrose	625	12.4653, 0	201	1.289070817	0.366331523
17	Mannose	716	17.7116, 0	160	1.283416172	0.359989067
18	(+)-catechin	770	25.943, 0	368	1.273170136	0.348425222
19	Arachidic acid	696	22.7363, 0	117	1.248480747	0.320173573
20	Myristic Acid	721	17.3481, 0	117	1.235081798	0.304606593
21	Gentiobiose	761	25.6645, 0	159	1.216287179	0.282483905
22	Caprylic acid	565	10.2849, 0	117	1.203475382	0.26720663
23	Linoleic acid methyl ester	693	19.7872, 0	67	1.172126408	0.229128166
24	Pantothenic acid	595	18.7426, 0	103	1.15867036	0.212470181
25	Allose	510	14.9788, 0	73	1.139934375	0.188950772
26	Glucose-1-phosphate	834	16.4356, 0	217	1.124644616	0.169469187
27	3,4-Dihydroxybenzoic acid	691	17.0211, 0	193	1.078014942	0.108377175
28	Palmitic acid	921	19.3057, 0	117	1.077804088	0.108094964
29	24,25-D	732	29.5233, 0	129	1.077594124	0.107813889
30	Methyl Phosphate	744	9.03833, 0	241	1.071372809	0.099460587
31	Lactobionic Acid	727	25.1029, 0	103	1.06437028	0.090000132
32	Oxalic acid	744	8.37214, 0	147	1.038639949	0.054695622
33	Succinic acid	915	10.9433, 0	147	1.033169414	0.047076839
34	Phenylalanine	758	14.0302, 0	120	1.033121867	0.047010444
35	Heptadecanoic acid	588	20.2102, 0	117	1.031510429	0.044758407
36	Canavanine degradation product	653	9.68057, 0	73	1.030270126	0.043022646
37	D-erythro-Sphingosine	712	22.5749, 0	204	1.020578213	0.029386749
38	Stearic acid	923	21.0941, 0	117	1.013022356	0.018666013
39	Purine riboside	627	22.1152, 0	204	1.01128078	0.016183614
40	Glycolic acid	858	7.60929, 0	147	1.006643287	0.009552542
41	Xylose	668	15.1727, 0	58	0.99408329	−0.008561361
42	Citric acid	899	16.9872, 0	273	0.990982466	−0.013068564
43	Levoglucosan	815	15.7427, 0	73	0.971380984	−0.041890851
44	Adipamide	591	15.4249, 0	73	0.969640249	−0.04447851
45	Lactic acid	896	7.383, 0	147	0.920221145	−0.119947487
46	Conduritol b epoxide	822	18.366, 0	217	0.915967034	−0.126632419
47	Galactinol	877	26.6601, 0	204	0.913701213	−0.130205624
48	4-Hydroxypyridine	548	8.78174, 0	152	0.90329289	−0.146734242
49	Fructose 2,6-biphosphate degradation product	658	20.77, 0	227	0.89848769	−0.154429357
50	Phosphate	883	10.3555, 0	314	0.884975422	−0.176290707
51	Zymosterol	782	28.7493, 0	129	0.880459836	−0.183670901
52	Cycloleucine	619	11.6332, 0	156	0.867422618	−0.205193034
53	3-Hydroxypyridine	690	8.54185, 0	152	0.864681507	−0.209759261
54	Shikimic acid	711	16.8845, 0	204	0.863287648	−0.212086749
55	2-Hydroxypyridine	899	7.11652, 0	152	0.856290872	−0.223827149
56	Dioctyl phthalate	718	23.5127, 0	149	0.85394986	−0.227776731
57	Melezitose	672	30.6607, 0	73	0.849834227	−0.234746645
58	Neohesperidin	631	23.0325, 0	73	0.84795516	−0.237940118
59	Alpha-ketoglutaric acid	552	14.2693, 0	73	0.839053084	−0.253166007
60	3-Hydroxypropionic acid	517	8.52757, 0	147	0.836665188	−0.257277687
61	Xylitol	773	15.6522, 0	103	0.811904201	−0.300618584
62	6-Phosphogluconic acid	656	22.4725, 0	318	0.807649498	−0.308198764
63	D-(glycerol 1-phosphate)	808	16.3675, 0	299	0.789007599	−0.341888899
64	Sorbitol	875	18.1171, 0	205	0.78056824	−0.357403332
65	Ethanolamine	885	10.294, 0	174	0.76322936	−0.389811424
66	Cytidine-monophosphate degradation product	681	22.7974, 0	217	0.753914289	−0.407527578
67	N-Acetyl-beta-D-Mannosamine	587	19.8309, 0	73	0.751812419	−0.411555347
68	Oxoproline	913	13.6434, 0	156	0.749449211	−0.416097382
69	Tagatose	558	17.1171, 0	159	0.738433894	−0.437459322
70	Glycine	815	10.8362, 0	174	0.712376746	−0.489287672
71	Galactonic acid	680	18.754, 0	73	0.708858446	−0.496430535
72	Melibiose	772	26.0418, 0	103	0.708279326	−0.497609663
73	Threitol	735	13.3288, 0	73	0.706837158	−0.500550212
74	O-Phosphorylethanolamine	648	16.6066, 0	174	0.692607292	−0.529890519
75	Sucrose	773	24.218, 0	236	0.685175164	−0.545455237
76	Beta-Alanine	831	12.4519, 0	174	0.609113861	−0.715216161
77	L-Allothreonine	796	11.879, 0	117	0.597106079	−0.743940839
78	Proline	559	10.7269, 0	142	0.592954073	−0.75400773
79	4-Aminobutyric acid	898	13.7383, 0	174	0.565448419	−0.822532668
80	Myo-inositol	914	19.6744, 0	217	0.509369384	−0.973215848
81	Alanine	923	7.98757, 0	116	0.503439525	−0.990109607
82	Aminomalonic acid	595	12.9704, 0	73	0.403634735	−1.308877763
83	Aspartic acid	895	13.5985, 0	232	0.388960505	−1.362304422
84	Isoleucine	768	10.6534, 0	158	0.370717731	−1.431606975
85	2-Monoolein	610	24.8258, 0	103	0.23096137	−2.114276523
86	Hydrocortisone	574	26.703, 0	96	0.000106275	−13.19990792
87	Carbamoyl-aspartic acid	595	16.7499, 0	257	0.000010876	−16.49729395

**Table 4:** List of factors affecting metabolic pathways.

Serial Number	Pathway
1	Glycine, serine, and threonine metabolism
2	Lysine biosynthesis
3	beta-Alanine metabolism
4	Alanine, aspartate, and glutamate metabolism
5	alpha-Linolenic acid metabolism
6	Valine, leucine, and isoleucine biosynthesis
7	Cysteine and methionine metabolism
8	Valine, leucine, and isoleucine degradation
9	Arginine and proline metabolism
10	Cyanoamino acid metabolism
11	Aminoacyl-tRNA biosynthesis
12	Methane metabolism
13	Vitamin B6 metabolism
14	Nicotinate and nicotinamide metabolism
15	Nitrogen metabolism
16	Pentose phosphate pathway
17	Carbon fixation in photosynthetic organisms
18	Glutathione metabolism
19	Biosynthesis of unsaturated fatty acids
20	Glucosinolate biosynthesis

**Table 5:** Details of the differential metabolites and metabolic pathways.

Number of Differential Metabolites Involved in a Metabolic Pathway	Name of Differential Metabolites
Four and above (13)	Isoleucine, leucine, threonine, glutamine, valine, aspartic acid, lysine, serine, alanine, glycine, carbon dioxide, pyruvate, glutamic acid
Three (13)	Cysteine, methionine, D-glyceraldehyde 3-phosphate, D-ribose 5-phosphate, spermidine 3-methyl-2-oxopentanoic acid, alpha-ketoisovaleric acid, 2-oxo-valeric acid, 4-methyl ester, L-aspartyl-4-phosphate, high threonine, tetrahydrofolate, 2-ketobutyrate, tryptophan
Two (23)	3-phosphate-D-glyceride, 5,10-methylene-THF, L-aspartic acid-semialdehyde, L-leucyl-tRNA, L-isoleucyl-tRNA, L-Valyl-tRNA, asparagine, phenylalanine, arginine, proline, D-glyceraldehyde 3-phosphate, dihydroxyacetone, heptapheptaphosphate, D-glyceraldehyde 3-phosphate, D-ribose 5-phosphate, D-erythritose 4-phosphate, (S)-1-pyrrolidin-5-carboxylate, fumarate, γ-aminobutyrate, aarbamate phosphate, α-linolenic acid, 2-oxo-4-methylthiobutyrate, S-adenosylmethionine amine

**Table 6:** A breakdown of the division of the Heilongjiang temperate zone.

Accumulated Temperature Zone	Active Accumulated Temperature	Area
First accumulated temperature zone	Above 2700 °C	Qiqihar city, Fularki district, Ang’angxi district, King Star town, Tailai county, Dorbod Mongol Autonomous county, Harbin city, Bin county, Acheng district, Shuangcheng district, Hulan district, Daqing city, Datong district, Zhaoyuan county, Zhaodong city, Zhaozhou county, Dongning city, Sanchakou town.
Second accumulated temperature zone	2500–2700 °C	Longjiang county, Gannan county, Beidahuang Shuanghe, Fuyu county, Fulu town, Longanqiao town, Lindian county, Dawn village, Daqing city, Mudanjiang city, Hailin city, Ning’an city, Langone, Bayan county, Mulan county, Wuchang city, Fangzheng county, Yilan county, Suihua city, Qinggang county, Wangkui county, Lanxi country, Jiamusi city, Huanan county, Boli county, Tangyuan county, Huachuan county, Jixi city, Jidong county, Mishan city, Beidahuang 857, Beidahuang 857, Beidahuang Xingkaihu, Jixian county, Fujin city, Beidahuang Youyi, Beidahuang Hongxinglong, Baoqing county, Beidahuang 291.
Third accumulated temperature zone	2300–2500 °C	Nehe city, Yi’an county, Keshan county, Baiquan county, Mingshui county, Suiling county, Qing’an county, Beidahuang Liuhe, Shuangyashan city, Beidahuang 853, Beidahuang 852, Qitaihe city, Linkou county, Muling city, Yanshou county, Shangzhi city, Tonghe county, Hegang city, Beidahuang Baoquanling, Jixi Lishu district, Hulin city, Beidahuang Qingfeng, Tongjiang city, Beidahuang Jiansanjiang, Beidahuang Daxing.
Fourth accumulated temperature zone	2100–2300 °C	Nenjiang city, Beidahuang Jiusan, Beidahuang Heshan, Beidahuang Lantau peak, Beidahuang Hongwuyue, Beidahuang Rongjun, Beidahuang Zhaoguang, Beidahuang Hailun, Beidahuang Honggunang, Bei’an city, Kedong county, Hailun city, Tieli city, Wudalianchi city, Yabuli forestry bureau, Weihe Forestry Bureau, Yichun city, Beidahuang Qinglongshan, Beidahuang Qianjin, Beidahuang Chuangye, Beidahuang Hongqiling, Beidahuang Shengli village, Heihe city, Xunke county, Jiayin county, Changsheng, Beidahuang 855, East red town, Beidahuang Yunshan, Luobei county, Raohe county.
Fifth accumulated temperature zone	1900–2100 °C	Beidahuang Jianbian, Beidahuang Nenbei, Beidahuang Shanhe, Beidahuang Qixingpao, Beidahuang Mount erlong, Zhanhe forestry bureau, Beidahuang Hongxi, Huma county, Sunwu county, Beidahuang Qindeli, Beidahuang Qianfeng, Beidahuang 859, Fuyuan county, Yichun city, Malin forest farm, Sifangshan forest farm, Suifenhe city.
Sixth accumulated temperature zone	Below 1900 °C	Daling forest farm, Zhanbei forest farm, Chenqing town, Beidahuang Longmen, Beidahuang Changshuihe, Greater higgnan mountains.

**Table 7:** Instrument parameters.

Project	Parameter
Sample volume	1 μL
Front inlet mode	Splitless Mode
Front inlet septum purge flow	3 mL/min
Carrier gas	Helium
Column	DB-5MS (30 m × 250 μm × 0.25 μm)
Column flow	1 mL/min
Oven temperature ramp	50 °C held for 1 min, raised to 310 °C at a rate of 10 °C/min, held for 8 min
Front injection temperature	280 °C
Transfer line temperature	280 °C
Ion source temperature	250 °C
Electron energy	−70 eV
Mass range	*m*/*z*: 50–500
Acquisition rate	12.5 spectra per second

## Data Availability

Not applicable.

## Sample Availability Statement

Samples of the compounds are not available from the authors.
